# Pregnancy Incidence and Correlates in a Clinical Trial Preparedness Study, North West Province South Africa

**DOI:** 10.1371/journal.pone.0095708

**Published:** 2014-05-06

**Authors:** Candice M. Chetty-Makkan, Katherine Fielding, Paul J. Feldblum, Matt A. Price, Petra Kruger, Heeran Makkan, Salome Charalambous, Mary H. Latka

**Affiliations:** 1 The Aurum Institute, Rustenburg, South Africa; 2 London School of Hygiene and Tropical Medicine, London, United Kingdom; 3 FHI 360, Durham, North Carolina, United States of America; 4 International Aids Vaccine Initiative, New York, New York, United States of America; 5 Department of Epidemiology and Biostatistics, University of California San Francisco, San Francisco, California, United States of America; University of Southampton, United Kingdom

## Abstract

**Introduction:**

Women in HIV prevention trials often must typically agree to avoid pregnancy. Regardless, some become pregnant. Screening tools predicting pregnancy risk could maximize trial safety and efficiency.

**Objectives:**

We assessed incidence and correlates of pregnancy among women at high HIV risk.

**Methods:**

We enrolled sexually-active, HIV-negative women into an observational cohort (2008–2011). At enrolment demographic, contraceptive, reproductive, pregnancy intention and behavioural data were collected. Women reported if one or both partners wanted or intended for the couple to become pregnant. We measured gender role beliefs using a locally validated eight-point index. We tested HIV and pregnancy, and inquired about sexually transmitted infection symptoms (STIs) at enrollment and monthly. HIV testing included behavioural counselling and condom provision, but did not specifically counsel women to avoid pregnancy. Cox proportional hazard modelling evaluated the associations with pregnancy. The multivariate model included the following variables “Recent pregnancy attempts”, “Gender Roles Beliefs”, ”Self-reported STIs” and “Age”.

**Results:**

We screened 1068 women and excluded (24.6%, 263/1068) who did not report risk behaviour. Non-pregnant, non-sterilized women aged 18–35 (median = 21 years) enrolled (n = 438). Most women reported one partner (74.7%) and a prior live birth (84.6%). Median follow-up time was 6 months (range 0.7–15.5). Pregnancy incidence was 25.1 per 100 women-years (n = 57 pregnancies). Conservative beliefs on gender roles (Adjusted Hazard Ratio (aHR) 1.8; 95% confidence interval [CI] 1.1–2.9), recent pregnancy attempts (aHR 1.9; 95% CI 1.1–3.4) and baseline self-reported STI (aHR 2.5; 95% CI 1.4–4.4) were associated with increased incident pregnancy. Report of no pregnancy intention was associated with lowered pregnancy risk (aHR 0.3; 95% CI 0.1–0.7).

**Conclusions:**

We identified new and confirmed existing factors that can facilitate screening for pregnancy risk.

## Introduction

As the HIV pandemic continues, 2.5 million new infections were reported worldwide in 2011, 59% of them in sub-Saharan women, which underscores the need to develop HIV prevention strategies for women [1], [2]. Much of the pandemic is concentrated in sub-Saharan Africa, especially Southern Africa and women are more likely than men to be infected (22.5% vs. 13.1%) [3]–[4]. Majority of recent HIV prevention trials commonly enrol women [5]–[6].Therefore, the participation of women in HIV prevention trials generally, and in South Africa in particular, is crucial.

Intended pregnancy rates in the developing world (85 pregnancies per 1000 women aged 15–44 years) are higher when compared to the developed world (48 pregnancies per 1000 women aged 15–44 years) [33]. Africa also had the highest rates of intended and unintended pregnancies (136 and 86 pregnancies per 1000 women aged 15–44 years). Differences within Africa vary by region: 140, 147 and 243–263 pregnancies per 1000 women aged 15–44 years in Southern Africa, Northern and Central/Western Africa [33]. An emerging challenge in HIV prevention clinical trials is the need to enrol women at higher risk of HIV, but these same women may also be at high risk for pregnancy. Further, trials must minimize pregnancy incidence during follow-up to maximize safety of mother and child [7]. When an unintended pregnancy occurs during clinical vaccine trials the toxicology impact of the vaccine could have a negative effect on mother and developing foetus that cannot be removed. During microbicide trials, when an intended pregnancy occurs, use of the test product is usually discontinued as pregnancy safety data may not always be immediately available [9]. Current practice for pregnancy risk screening in HIV prevention clinical trials is a verbal close ended report of intent to become pregnant. This often takes place after widespread community education about the requirements for trial entry that includes avoiding pregnancy whilst enrolled.

Despite women stating that they do not intend to become pregnant, pregnancy incidence within clinical trials ranges between 3.1–20.2/100 women-years [WY]) [8]–[14], [31]. In pre-trial feasibility studies, where women were requested to avoid pregnancy and provided with family planning counselling, condoms and/or hormonal contraceptives, the incidence of pregnancies remained high (17.4–20.2/100 WY) [15]–[19]. Therefore, solely asking women about their intent to avoid pregnancy while enrolled in a trial may not provide a true measure of pregnancy intent. Women who screen for a trial may have already been educated about requirements such as avoiding pregnancy and choose to report no pregnancy intent during screening due to perceived study benefits [32]. Alternate methods for assessing pregnancy risk within HIV prevention trials are needed.

Six African HIV prevention trials have investigated risk factors for pregnancy [8], [10], [12]–[14], [17]. In these trials, women most at risk for incident pregnancy were single, younger than 25 years, engaged in unprotected sex in the week prior to enrolment, had more than one past pregnancy, did not use contraceptives or used them inconsistently, knew the HIV status of their partner/s and used substances (marijuana or alcohol) [8], [10], [12]–[14], [17]. Use of hormonal or barrier methods of contraception, availability of effective contraception onsite, and becoming HIV positive while enrolled in a trial lowered pregnancy risk [8], [10], [14], [17]. Thus, while there is growing literature on individual and situational factors associated with pregnancy risk, screening out these individuals prior to trial enrolment remains a challenge as evidenced by pregnancy rates during trials.

In order to find better screening measures for pregnancy risk we used epidemiological and social science literature to evaluate variables that were important to pregnancy risk in an observational study where women were not required to use contraception. The main aim of this study was to identify a profile that was associated with short-term pregnancy risk that could be used to inform and improve pregnancy screening methods in future prevention trials.

## Methods

The study took place in Rustenburg, North West Province (NWP) in South Africa. This is a prominent platinum mining town with an estimated population of 400 00 [20]. Data from population surveys conducted during 2009 and 2011 showed that literacy levels among women were reported to be low where 20% completed high school and only 5% received some form of tertiary education. The estimated unemployment rate was approximately 40% with over half (53.1%) of the unemployed being women [20]–[21].

### Ethics statement

We obtained approval for the study from the Biomedical Research Ethics Committee, University of KwaZulu-Natal, FHI 360 Institutional Review Board (IRB) and the research committee of the North West Provincial Department of Health. We obtained voluntary written informed consent from all participants in their preferred language and in the case of illiterate participants, a thumbprint to acknowledge understanding in the presence of a witness.

### Parent studies

From 2008 to 2011, we conducted a series of prospective cohort studies (Family Health International (FHI) and International Aids Vaccine Initiative (IAVI)) to estimate the HIV incidence in Rustenburg. The primary purpose of the FHI study was to estimate HIV incidence among a high risk population of women in Rustenburg, North West Province, South Africa. The duration of the FHI study was from 30 October 2008–01 December 2009. The primary purpose of the IAVI study was to measure the annual incidence of HIV infection, describe HIV-related epidemiology and selection biases among the screened and enrolled samples; characterize early HIV infection, and prepare this clinical trial site for HIV preventive vaccine efficacy trials for which participants from this study cohort may be recruited. The duration of the study was from 2 December 2009–12 December 2011.

Over this period we implemented two protocols, from different funding sources. These protocols were designed to select women at higher risk of HIV and who engaged in high risk sexual activity (i.e. those reporting multiple partners, STI symptoms and unprotected sex). Women who were not sexually active three months prior to enrolment were excluded. Neither study tested an investigational product so that women were not required or even counselled to avoid pregnancy. However, all women in the cohort were provided with behavioural counselling and condoms and referred for contraception on request. Women who were identified with STI symptoms at baseline were referred to the local clinic for treatment. We recruited participants from venues such as family planning and HIV testing services of local health clinics, educational institutions, and targeted community areas. Women were eligible for the cohort studies if they were between the ages of 18 and 49 years, able to provide voluntary informed consent, agreed to HIV and urine pregnancy testing, tested negative for HIV at screening, were not planning to relocate in the next six months, and reported high-risk sexual activity. All participants received financial compensation at each scheduled visit and gave their written informed consent prior to data collection. The participants were reimbursed R50-00 (South African rand, approximately 4–5 USD) per visit for their time and participation.

At enrolment, trained social scientist interviewers administered a structured questionnaire that took an average of 30 minutes to complete. Items included demographics, contraceptive and reproductive history, HIV-related risk factors and sexual behaviour. Participants provided information on behavioural activities that took place three months prior to enrolment, including participants' past pregnancy attempts. Data collected for the categories summarised above include information on language acculturation, pregnancy history, parity, contraceptives use, any (male/female) condom use, the total number and HIV status of sexual partners. This questionnaire was updated in July 2010 when we added a question to measure self-report intention to become pregnant in the 12 months after enrolment. Therefore only, a sub-set of the cohort provided data for this question. During all study visits, we used a clinician-reviewed checklist to verbally screen for sexually transmitted infection (STI) symptoms.

At screening and for monthly visits, participants were tested for HIV using rapid test. Pregnancy tests were conducted monthly on urine samples using the Quickvue One-Step hCG Combo test. The test has a>99% sensitivity and >99% specificity concordance rating. Both internal and external quality control systems were used to maintain testing compliance. Women who became pregnant during the study continued with follow-up visits where their pregnancy status was confirmed over time.

### Study data set for analysis

The data from the parent studies described above were pooled as the eligibility criteria for cohort entry, testing, methods, staff, and follow-up procedures were similar. To be eligible for this analysis, women had to be within peak fertility age range of 18–35 years, have no history of sterilization or hysterectomy, HIV negative, not pregnant at enrolment, and sexually active in the last three months prior to enrolment. Date of cohort entry was set at 13 November 2008.We included follow-up data until 12 December 2011. For the analysis participants were censored at the earliest of date of first positive pregnancy test or last follow-up visit. Enrolled women with no follow-up data (n = 65) were excluded. Follow-up for the cohorts were stopped due to funding constraints and revisions in the protocol target populations.

We measured beliefs on gender roles using an eight-item index with Likert four-level response options, derived and previously validated from research conducted in South Africa [22]. The estimate of the internal consistency (Cronbach's alpha) of the scales was reported 0.73. Responses across all eight items were summed for each respondent, which we used as an index of how progressive or conservative each woman was with respect to gender roles. This index included statements such as “Men have many lovers because it is in their nature to do so”, and “Women who are financially independent do not want to commit themselves to one relationship”. We scored participants' beliefs on their gender role within relationships as conservative (≤16), neutral (17–23) or progressive (≥24) [22].

Past pregnancy attempts was measured by the women reporting if one or both partners wanted the couple to become pregnant. Pregnancy attempt in the three months prior to screening was collapsed into a binary variable (both or either wanted to get pregnant versus no pregnancy attempt), due to sparse data. We did not conduct physical examination or laboratory testing for STIs. Instead, a nurse or doctor, administered an eight-item symptom checklist, and then made a clinical judgment on whether there were signs of an STI symptom based on participant's verbal responses. Participants were categorized as having STI symptoms if in the opinion of the clinician they reported at least one symptom of an STI, or reported diagnosis or treatment of STI symptoms in the last three months prior to enrolment [30].The outcome measure was incident pregnancy, defined as the first positive pregnancy test during follow-up.

We used STATA version 12.0 for the analysis. To describe the enrolled sample we used median and interquartile range (IQR) for continuous variables and frequencies and percentages for categorical variables. Pregnancy rates were reported as number of pregnancies per 100 woman years (WY) with an associated 95% confidence interval (CI). We used Cox proportional hazards regression to estimate the bivariate hazard ratios (HRs) and 95% CIs for time to pregnancy. As the number of pregnancies was small we limited the adjusted analysis to a model with a maximum of four parameters (hazard ratios) [23]. All variables in the unadjusted analysis which had P-values <0.10 were considered for the multivariable analysis, as well as age group, *a priori*. After adjustment, those variables with P-values <0.10 were retained in the model. All P-values are calculated from the likelihood ratio test. Regarding the sub-set of women who were administered the questionnaire with the question about pregnancy intentions, we evaluated the unadjusted association between pregnancy intent and pregnancy incidence, as the modest sample size did not support multivariable analysis.

## Results

**Figure 1:**
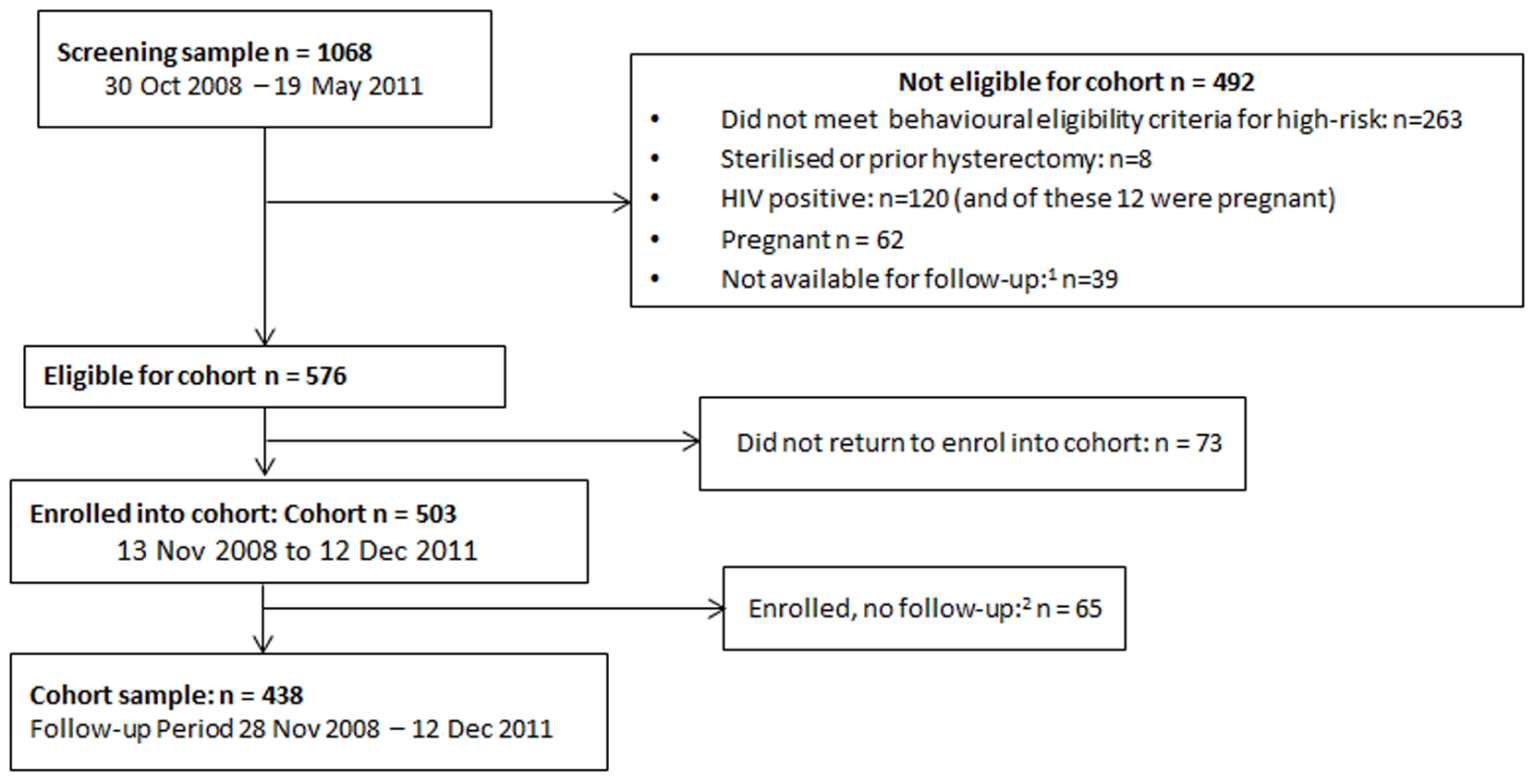
Flowchart to derive analytic sample. ^1^ n = 38 relocating and n = 1 refused follow-up ^2^ Follow up period was cut short for administrative reasons (funding halted) so 26 of the 65 women contributed no follow up time.

From 1068 women screened, (n = 576) 53.9% were eligible for the cohort, of which (n = 503) 87.3% had enrolled into the cohort (Figure 1). Among those screened out, (n = 263) 53.5% did so for not reporting what we deemed to be high risk behaviours. Among enrolled women, (n = 65) 12.9% did not have any follow-up time (premature closure of the study) and were excluded from the analysis, leaving 438 women in our analysis.

Median age at enrolment was 21 years (IQR 20–24 years), and most women were unemployed (68.9%) (Table 1). Three-quarters of women reported past pregnancy attempts (76.3%). Most women reported having at least one prior live birth (84.6%). Among the 230 women classified with STI symptoms according to our definition at baseline, 8.2% were diagnosed and 17.8% treated for STIs in the past 3 months, while 74.0% self-reported at least one current symptom at baseline. The majority of women reported having one partner (74.7%) and no new partner in the past three months (70.3%). Regarding beliefs on gender roles, (14.8%) of women held conservative views on gender roles, (69.2%) neutral and (15.9%) were classified as progressive.

**Table 1:** Cohort description, pregnancy rate and associations with pregnancy incidence in HIV negative women (n = 438; 57 pregnancies).

Independent Variables	Sample Characteristics (%)	Total pregnancies (n)	Time to follow-up (Person Years)	Rate per 100 person-years (No. pregnancies/wyrs)	Crude Hazard Ratio	p-value
**Age**						
18–20 years	178 (40.6)	18	0.9	19.9	1	
21–25 years	185 (42.2)	31	1.0	31.5	1.5 (0.8–2.7)	0.144
26–35 years	75 (17.1)	8	0.4	20.5	0.9 (0.4–2.2)	0.953
**Employment**						
Unemployed	302 (68.9)	38	1.5	24.6	1	0.866
Employed	136 (31.1)	19	0.7	26.1	1.0 (0.6–1.8)	
**Language Acculturation^1^**						
English	207 (47.4)	24	1.1	21.5	1	
Setswana	133 (30.4)	17	0.7	25.1	1.2 (0.6–2.2)	0.653
Mix of English and Setswana	97 (22.2)	16	0.5	33.3	1.6 (0.9–3.1)	0.138
**Past pregnancy attempt in 3 months prior to enrolment^2^**						
No	312 (76.3)	36	1.7	21.4	1	0.022
Yes (Participant perceived either 1 or both partners in the relationship as trying to become pregnant)	97 (23.7)	18	0.4	40.7	1.9 (1.1–3.4)	
**Parity^3^**						
0 or 1 live birth	297 (84.6)	41	1.5	27.9	1	0.382
2 or more live births	54 (15.4)	6	0.3	19.6	0.7 (0.3–1.6)	
**Any use of hormonal contraception in the 3 months prior to enrolment^4^**						
No	272 (75.9)	44	1.4	31.2	1	0.260
Yes	86 (24.0)	7	0.4	18.7	0.6 (0.3–1.4)	
**Any use of male or female condoms in 3 months prior to enrolment with main partner^5^**						
No	133 (39.8)	18	0.7	26.1	1	0.874
Yes	201 (60.2)	24	1.0	24.3	0.9 (0.5–1.8)	
**Induced Abortion in the 3 months prior to enrollment^6^**						
No	412 (97.9)	53	2.1	24.7	1	0.672
Yes	9 (2.1)	2	0.1	34.5	1.4 (0.3–5.6)	
**Self-reported symptoms, diagnosis or treatment of sexually transmitted infections in the 3 months prior to enrolment**						
No	208 (47.5)	21	1.2	16.9	1	0.001
Yes	230 (52.5)	36	1.0	34.9	2.6 (1.5–4.6)	
**Total number of sex partners in the 3 months prior to enrolment**						
None	20 (4.6)	3	0.1	31.2	1	
One	327 (74.7)	39	1.7	22.4	0.7 (0.2–2.1)	0.479
Two or more sex partners	91 (20.8)	15	0.4	34.1	1.0 (0.3–3.5)	0.979
**Total number of new partners in 3 months prior to enrolment**						
No new sex partner	308 (70.3)	38	1.7	22.5	1	0.139
1 or more	130 (29.7)	19	0.6	32.3	1.5 (0.9–2.6)	
**Total number of HIV positive partners^7^**						
None	122 (93.1)	21	0.5	41.6	1	0.545
1 or more	9 (6.9)	3	0.1	57.8	1.5 (0.4–4.9)	
Gender Roles Beliefs*						
Progressive View (1)	70 (15.9)	6	0.4	15.7	1	0.039*
Neutral View (2)	303 (69.2)	39	1.6	24.5	1.7 (1.0–2.7)	
Conservative View (3)	65 (14.8)	12	0.3	39.7	2.7 (1.1–7.1)	

Footnotes: *Assuming a linear dose response where gender roles beliefs coded as progressive view = 1 neutral = 2, conservative view = 3 ^1^n = 1 participant who spoke isiXhosa was removed from the analysis; ^2^This was the female participant's perception. n = 29 unknown, not applicable and missing data; ^3^n = 87 unknown, not applicable and missing data; ^4^n = 80 unknown, not applicable and missing data; ^5^n = 104 unknown, not applicable and missing data; ^6^n = 17 unknown, not applicable and missing data; ^7^n = 307 unknown, not applicable and missing data.

The cohort was followed for a median of six months (range 0.7–15.5) during which 57 pregnancies occurred over a total of 227.5 WY, giving a pregnancy rate of 25.1 per 100 WY (95% CI 19.3–32.5; Table 1). There were no multiple pregnancies and the 57 pregnancies occurred in 57 different women. Pregnancies were highest among women who were between the ages of 21–25 years (31.5/100 WY), reported past pregnancy attempts (40.7/100 WY), showed signs of STI symptoms (34.9/100 WY), and had a conservative view on gender roles (39.7/100 WY). In Table 1, we did not see a statistical difference in terms of contraceptive method when use and non-use of hormonal contraception (p>0.1) and hormonal barriers (p>0.1) were compared. We do not have contraceptive use data in follow-up, as data on contraceptive use was only collected at baseline. In the univariable analysis the following variables were significantly associated with increased pregnancy rate: past pregnancy attempts, STI symptoms, and more conservative gender roles beliefs. The final multivariable model showed that past attempt from either one or both partners in the relationship to become pregnant, having a conservative view on gender roles, and signs of STI symptoms were independently associated with increased pregnancy incidence (Table 2).

**Table 2:** Multivariable model of the association of past pregnancy attempts, gender roles beliefs, STI symptoms and age with pregnancy incidence (n = 409; 54 pregnancies)^1^.

	Adjusted Hazard Ratio (95% CI)**	P-value*
**Past pregnancy attempt in 3 months prior to enrolment ^2^**		
No attempt to become pregnant	1	0.032*
Any attempt to become pregnant	1.9 (1.1–3.4)	
Gender Roles Beliefs^3^		
Progressive View	1	0.011*
Neutral View	1.8 (1.1–2.9)	
Conservative View	3.3 (1.3–8.4)	
**Self-reported symptoms, diagnosis or treatment of sexually transmitted infections in the 3 months prior to enrolment**		
No	1	0.002*
Yes	2.5 (1.4–4.4)	
**Age**		
18–20 years	1	0.087
21–25 years	1.8 (1.0–3.4)	
26–35 years	1.0 (0.4–2.5)	

Footnotes: ^1^Number of observations reduced to 409 and number of pregnancies 54 due to missing data on the trying to become pregnant variable. ^2^This was the female participant's perception. n = 29 unknown, not applicable and missing data. ^3^Test for linear trend; relationship belief coded as progressive view = 1, neutral = 2, conservative view = 3. * From likelihood ratio test. ** All variables have been adjusted for each other.

In the sub-sample of women (n = 184) who were asked the question on intention to become pregnant, 21 pregnancies occurred. In unadjusted analysis couples with intention for pregnancy were more likely to become pregnant, compared with couples who reported no intention for pregnancy (i.e. one or both members of the couple intended to become pregnant; HR 3.6 [95% CI 1.5–8.6]). The small sample size precluded further analysis.

## Discussion

Pregnancy rates within this study were high when compared to other feasibility studies that were conducted in Africa [15]–[19]. We identified three risk factors associated with an increased risk of falling pregnant. Two of these risk factors were a prior history of trying to become pregnant at study entry, and conservative beliefs about the role of men and women in relationships. These factors represent promising new ways of screening women for pregnancy risk in trials. Many potential trial participants may not be aware that their most recent reproductive attempts or their beliefs about gender roles could be interpreted as pregnancy risk. Therefore women may be less likely to shape their self-report on these aspects simply to gain access to the trial.

Screening women out on these factors may help trialists assemble a cohort of trial participants at truly lower risk of pregnancy, minimizing the potential of risk to mother and potential foetus. These two risk factors also have the added advantage of being self-reported and short and simple to administer. Also, to improve efficiency, the gender role index can be readily hand-calculated in real-time. These measures also do not rely on laboratory test results and therefore can be used as a first pre-screening step to maximize trial efficiency. This could ensure that only the most promising candidates for the trial are sent on for more intensive, costly and invasive procedures.

The gender beliefs scale was locally validated and applicable to women within the research setting [22]. Data collected during a representative household survey in Rustenburg, South Africa were used to validate this scale [22]. Therefore the questions used and the findings of this study are generalizable to this community, but is still unknown whether findings apply beyond this context. Conservative gender role beliefs imply that a woman defines herself in terms of motherhood, fidelity, economic dependence and being submissive in a relationship [22], [24]–[26]. Thus, adhering to such cultural expectations and a desire to fulfil their maternal desire or role would likely increase pregnancy risk. We also found that women in their early twenties were more likely to become pregnant compared with their younger peers. Age has been found as a pregnancy risk factor in other studies [8], [14]. It is possible that cultural expectations for women may also be operating to increase the pregnancy rate among this particular age group [4]. This finding on a high-risk age for pregnancy was observed independent of gender beliefs, so women in their early twenties who have a conservative view on gender roles who choose to enrol in clinical trials may need extra support for avoiding pregnancy.

The influence that partners' have on the behaviour of participants are also becoming increasingly important to pregnancy risk. Past studies show that pregnancy rates were increased when the partner's HIV status was known to be negative [10]. For this analysis, from the past attempts at trying to become pregnant and the pregnancy intent variables, we were able to obtain the participants view of the influence that her partner had regarding decisions to become pregnant. A screening tool that assesses beliefs on gender role dynamics and the role of the participant's partner in decision making around pregnancy is vital. Women with a conservative view on gender roles and who perceive their partners as influencing the decision to become pregnant may be at high risk for pregnancy while enrolled in a trial.

Women who reported recent or current STIs had twice the rate of becoming pregnant. STI symptoms may be a proxy of being sexually active, and engaging in unprotected sex which both put women at a higher risk of pregnancy. However, some STIs are relatively asymptomatic and could be a poor proxy. Nevertheless, STIs are highly prevalent in developing countries. Prevalence of STIs in South Africa has been reported to be between 20–40% for candidiasis and bacterial vaginosis, 10%–42% for syphilis and 5–31% for gonorrhoea [27], compared to our self-reported prevalence of just over 50% in the past three months for signs, symptoms, diagnosis or treatment of any STI. However, while STIs were poorly measured in the current study, this factor still emerged as being highly associated with pregnancy risk. For this study we also had a high screen out rate and inclusion was based on report of high-risk behaviour, therefore with this analytic sample we may have selected a group with a high prevalence of STI symptoms.

Women in our study were not required to avoid pregnancy, were not counselled to avoid pregnancy, nor provided with an on-site contraceptive service beyond condom provision, which may have helped to elevate the pregnancy rate we observed. Although we did provide referrals for contraception, we did not collect follow up data on these referrals, and thus could not consider this in our analyses. In the sub-group of women who were asked about their intention to become pregnant at screening, we found that intention was positively associated with pregnancy. This is similar to the literature where women with intended conceptions recognized early signs of pregnancy, sought prenatal care and engaged in less risky behaviour while pregnant [28]–[29]. However, within the context of a clinical trial, the utility of asking women about pregnancy intentions should be considered limited at best as most trials widely advertise throughout the community that women must avoid pregnancy to gain access to the trial.

The strengths of this study were the prospective design, evaluating a wide range of novel risk factors, and implementing the study in a neutral setting with respect to pregnancy expectations. A limitation is that certain behavioural questions were only measured at entry into the study and if participants changed the behaviour during the course of the study, this was not captured. We did not request participants to avoid pregnancy. Due to no restrictions on becoming pregnant, women within this reproductive age may not have found it necessary to avoid pregnancy. The small sample size of the sub group, inability to control for all risk factors from literature and lack of follow up data on pregnancy intention are limitations. Nonetheless, the aim was to identify effective, simple screening questions at early stage trial entry so our findings may contribution to better screening women during future clinical trials.

Based on our study, participants in their early twenties, who have a conservative view of relationships, report attempting to become pregnant prior to enrolment, and show signs of recent or current STIs should be considered at higher risk for pregnancy. In clinical trials that are enrolling participants where pregnancy would influence outcomes, one needs to consider excluding such patients from enrolment. Our data also suggest that the intentions and desires of the male partner (as measured from the perspective of the participant) may influence pregnancy risk. Reproductive counselling sessions before trial implementation should consider engaging the couple, not just the women. The counselling sessions should explore the couple's life stage, their stance on gender roles; and expectations as a way of potentially clarifying reproductive goals.
